# Saturated fat –a never ending story?

**DOI:** 10.1080/16546628.2017.1377572

**Published:** 2017-09-19

**Authors:** Karianne Svendsen, Erik Arnesen, Kjetil Retterstøl

**Affiliations:** ^a^ Department of Nutrition, Institute of Basic Medical Sciences, University of Oslo, Oslo, Norway; ^b^ The Norwegian Heart and Lung Association (LHL), Oslo, Norway; ^c^ Lipid Clinic, Medical Department, Oslo University Hospital, Oslo, Norway

**Keywords:** Saturated fat, fatty acids, cholesterol, coronary vascular disease, coronary heart disease, placebo, nutritonal sciences, low density lipoprotein

## Abstract

Science has no clear message regarding health effects of saturated fats, it seems. Different RCTs, prospective cohort studies and meta-analysis have led to contrasting conclusions. The aim of the present commentary is to discuss some possible reasons for an apparently never-ending fat controversy. They are of a purely scientific nature, which is important to recognize, but unfortunately hard to overcome. First is the placebo problem. In pharmaceutical science, evidence-based medicine is often synonymous with data on verified medical events from long-lasting double-blind randomized placebo controlled trials. In nutritional science the lack of double-blind design and lack of placebo food generate less conclusive data than those achieved in pharmaceutical science. Some scientists may apply the same type of scientific criteria used to evaluate the effects of drugs for foods. This leaves an impression of insufficient data since in this respect the fundamental criteria for evidence based medicine are not present. The next scientific problem is the energy balance equation. In contrast to pharmaceuticals, nutrients contain energy. An increased intake of one nutrient will lead to a decreased intake of another. The effect of change in only one nutrient is then difficult to isolate. Lastly, in nutritional science, generalizability is difficult compared to pharmaceutical science. Food culture interferes with lifestyle and food habits change over time. In conclusion, all available knowledge, from molecular experiments to population studies, must be taken in to account, to convert scientific data into dietary recommendations.

Saturated fatty acids (SFA) do not increase the risk of cardiovascular disease (CVD), two recently published analyses of randomized controlled trials (RCTs) concluded [1,2]. Replacing SFA with linoleic acid increased CVD risk in one trial [2]. Additionally, a recent large prospective cohort study reported lower CVD risk with increased intake of energy from SFA [3], and recent meta-analyses of cohort studies found that SFA did not increase the risk of CVD [4,5]. Such evidence supports and excites scientists and others who strongly argue for increasing the fat content of food, including SFA. However, counter-arguments referring to health benefits by reducing saturated- and trans-fatty acids are numerous, including recently published RCTs [6,7], prospective cohort studies [8–10] and ecological studies [11,12]. It seems as if science has no clear message regarding health effects of SFA despite an enormous amount of work over many years. The aim of the present commentary is to discuss some possible reasons for an apparently never-ending fat controversy.

## Introduction

A short commentary like this cannot elucidate the contradictions in depth, since each study must carefully be assessed, preferably by expert committees appointed by public authorities, without perceived intellectual or financial conflicts of interests, in order to avoid influence from various stakeholders. Many countries publish national dietary guidelines on a regular basis, and recently we reviewed nine national guidelines published from 2010 to 2016 for their recommendation on SFA [13]:

Eight out of nine guidelines recommended SFA intake to be 10 percent of total energy intake, or less.

The ‘devil’ is in the study details. For example, in a recent cohort study reporting possible benefits of SFA [3], the diet was assessed at study baseline in the mid-1990s and the participants were followed up to 25 years later, suggesting that assessment of the diet at one time-point can predict disease many years later, a very common study design. However, in another study with different results [9] the diet was measured eight times during a 25 years period. An interpretation that two rather similar studies deliver contrasting conclusions clearly ignores the very different methods used in these studies. Meta-analysis of such studies can at best deliver vague approximations since residual confounding is particularly important in nutrition science. The two recent studies mentioned above were extensively adjusted statistically but neither were adjusted, e.g. for the use of lipid lowering statins.

Errors like under- and over-reporting of dietary intake tend to be more severe with SFA camouflaged in a diversity of foods such as sauces and snacks in contrast to easily countable items like cups of coffee [14]. Few studies have analysed the associations with specific SFA, or their different food sources, which likely is a biologically relevant factor [9]. In addition, the between-subjects variation in dietary SFA is often limited, which precludes detection of significant outcome associations. As Rose stated more than 30 years ago: ‘The hardest cause to identify is the one that is universally present, for then it has no influence on the distribution of disease’ [15].

### The placebo problem

An important reason for the never-ending saturated fat controversy is probably a purely scientific issue which is important to recognize, but unfortunately hard to overcome. In nutritional science, all RCTs on hard endpoints, such as CVD events or mortality, suffer from the serious limitation of being neither placebo-controlled nor double-blind. In pharmaceutical science, evidence-based medicine is often synonymous with data from double-blind randomized placebo controlled trials (placebo-RCT) vital to obtain approval from regulatory authorities [16].

There are no possible placebo foods like placebo ham or steak that can be eaten for as many years as it takes to complete a study on hard endpoints. Without access to a real placebo, the double-blind design fails. The closest approach to a double-blind design on hard CVD endpoints in nutritional science is probably the Finnish Mental Hospital studies [17,18] and the Minnesota Coronary Experiment [1]. In these studies, hospitals were selected or randomized to serve foods low in SFA or no dietary change, which is far from a conventional placebo-RCT design. Even the largest dietary RCT to date, the Women’s Health Initiative Dietary Modification Trial [19], was not placebo-controlled. Advice on dietary fat reduction had no effect on CVD, but a post-hoc analysis showed a 19% lower risk in those who reduced their self-reported intake of SFA the most. Once again, there is doubt as to how this should be interpreted.

Scientists may claim that without any placebo-RCTs on CVD events or mortality, the fundamental criteria for evidence based medicine is not present. Clinicians may apply the same types of scientific criteria used to evaluate the effects of drugs, for foods. This will result in the impression of insufficient data on almost every food item due to the lack of placebo. Importantly, the lack of placebo-RCTs in nutritional science may not be sufficiently recognized by those who demand causal evidence from placebo-RCTs in the name of evidence-based medicine. The lack of definitive causal evidence for a hypothesis may be interpreted as a proof for its faultiness or an argument for an alternative hypothesis, e.g. that SFA has no effects on health [20].

Advocates of evidence-based medicine criticise the indirect evidence from observational data and may rely only on placebo-RCTs by default, but this is not without problems, as shown in the amusing parachute experiment [21]. Since the effect of parachutes is not documented by RCTs, participants were randomized to receive a real or a placebo parachute before jumping. Not surprisingly, it was hard to recruit healthy volunteers to this experiment. The lack of placebo-RCTs was also a major obstacle to prove the harmful effects of tobacco smoking. Obviously, the powerful tobacco industry used the lack of evidence for their benefit. Doubts on the science may be used for industry purposes. Impossible expectations for science is a characteristic of science ‘denialism’ [22]. Important financial and political stakeholders are involved in the sales and production of foods, with fats as no exception. Certainly, ‘big food’ may also profit from confusion and doubt about scientific results.

### The energy equation

In contrast to pharmaceuticals, macronutrients like fat contain energy. In an experimental, energy-controlled design, this implies that an increased intake of one nutrient will lead to a decreased intake of another nutrient. If fat is replaced by carbohydrate or protein, the isolated effect of fat will be influenced by a change in the other macronutrients to keep the diet iso-caloric, making the isolated effect of one macronutrient difficult to measure.

### The lack of studies on hard endpoints and the issue of generalizability of data

After a century of controversy on the role of cholesterol in atherosclerosis, finally a placebo-RCT showed that cholesterol-lowering statins reduced mortality [23].

At this time, in the 1990s, the effects of statins were proven in middle-aged males only; data for females wereas yet not statistically significant. Today, evidence clearly shows statins to be equally efficient in both sexes [24]. Nevertheless, some scientists argue against cholesterol as a risk factor for CVD [25] demonstrating that no matter how conclusive evidence is, it will always be questioned. Clearly, the debate on SFA will also be vibrant for years to come. Therefore, transparent assessment of quality of evidence is important. The Grading of Recommendations Assessment, Development and Evaluation (GRADE) is one useful approach to help evaluate the literature [26]. Using GRADE in evaluating RCTs Hooper et al. found ‘moderate’ quality evidence for the recommendation to replace SFA with polyunsaturated fats [27]. In observational studies, de Souza et al. found the evidence for a lack of association between SFA and CVD to be ‘very low’ [5].

## Conclusion

Nutritional science suffers from the lack of placebo-RCTs. Thus, to convert scientific data into dietary recommendations, all available knowledge, from molecular experiments to population studies must be interpreted. Knowledge syntheses by independent public scientific committees form the basis for national guidelines. Hence, a transparent assessment of the quality of the evidence base on which a conclusion is based should be provided. Despite several ongoing controversies like the apparently never-ending SFA controversy, the message from science to consumers from national guidelines is clear: Keep your intake of SFA low (below 10 energy percent) by replacing them with unsaturated fats and unrefined carbohydrates.
